# Effect of Menstrual Disorders upon the Vascularity and Nutrition of the Intra-ocular Structures

**Published:** 1872-06

**Authors:** Reuben A. Vance

**Affiliations:** New York City


*The Effect of Menstrual Disorders upon the Vascularity and Nutrition of the Intra-ocular Structures.* By Reuben A. Vance, M.D., New York City.

The careful observation of the intra-ocular structures with the ophthalmoscope, continued at short intervals for a length of time, will in certain cases reveal phenomena of great interest, both in a physiological and pathological point of view. This is especially true when the patients are women and the observations extend over such epochs as pregnancy and lactation, or menstruation and the climacteric period. We have then an opportunity of watching the effect upon the cerebral circulation of those bodily states which are notoriously competent to induce changes in the mental tone of the individual, and which, in certain cases, cause a most distressing form of insanity.

The phenomena of menstruation may be attended by disordered vision, and it is possible that such visual disorders are due to the general commotion to which the female organization is subjected at this time, yet such cases are rare; and Dr. T. Clifford Allbutt, of Leeds, in his recent work, says that he has never been able to satisfy himself of their existence.* I have seen a number of cases in which photophobia and dimness of vision were complained of at the monthly periods, in which the ophthalmoscope did not reveal any disorder of the intra-ocular structures, and others again where hyperæmia of the disk and retina to a very marked degree, occurring at the same time, was unattended by any defect of vision.

In very many cases the menstrual epoch is attended by great mental depression and occasionally by extreme bodily suffering. The mental vagaries, curious conceits and unnatural ideas and desires then developed are as diverse as the pains and aches which, at such times, are so commonly complained of by different patients. It will frequently happen that the peculiar tendency of the individual will then be developed, and an opportunity is thus afforded of learning more of the mental characteristics of a woman by a careful observation of her remarks and attention to her conduct during this period than at any other time. The details of a case which I presented in outline to the Medico-Legal Society of this city in December, 1871, and which was the subject of a memoir read before the Medical Journal and Library Association March 8th, 1872, illustrates this fact very clearly. A young woman who became the victim of morbid desires and vicious impulses at each menstrual period, but who was in perfect mental and physical health at all other times, gave no other evidence of cerebral dis-

* Allbutt, On the Use of the Ophthalmoscope in Diseases of the Nervous System and of the Kidneys; and also in certain other General Disorders. London: Macmillan & Co. 1871.

order than such as was revealed by the ophthalmoscope. While such peculiarities of disposition and conduct as were exhibited by this patient are among the rarities of medical experience, it is to be borne in mind that the physical state which initiated them in this case is very commonly induced by that peculiar condition of the system which occurs in connection with the phenomena of menstruation.

The ophthalmoscopic appearances in the majority of cases in which symptoms referable to the cerebro-spinal system have been observed, are such as denote an increase in the quantity of blood in the intra-ocular structures. The retinal circulation is affected, but not to the same extent as that of the disk. The vessels of the latter are enlarged and their number increased. It may even assume a crimson appearance, and I have occasionally seen it so congested that the whole fundus oculi appeared of a uniform color and the site of the papillæ could only be discovered by tracing the retinal vessels to their point of convergence. The arteries of the retina may be but slightly affected—as a rule they are not enlarged to any great extent—but the veins are increased in size and number, and their course becomes irregular and tortuous. It is the proper vessels of the disk which undergo the greatest change, and the chief evidences of congestion will be observed at the intra-ocular termination of the optic nerve.

In a small proportion of cases, yet cases in which the cerebral symptoms are of the same nature and equally severe with those of the former class, the ophthalmoscopic appearances are directly the reverse of those just described. Instead of hyperæmia of the disk and retina, we find an anæmic state of the intra-ocular structures. The lateral vessels of the disk disappear; the retinal arteries and veins are much smaller than in health—the former sometimes diminishing to a mere line; many branches previously visible now disappear from view, and the disk presents a clear, bloodless aspect, through which the openings in the lamina cribrosa can be plainly seen.

The proportion of cases in which intra-ocular congestion can be observed at the menstrual period is quite large, but the number of instances is small in which the ophthalmoscopic appearances of hyperæmia persist during the whole time the woman is unwell. In many cases where ladies suffering from menstrual irregularities have been under my care for disorders of the nervous system, it has been possible to pursue ophthalmoscopic observations for a length of time, and not only to see the intra-ocular appearances during the time they were menstruating, but also during the intervening period. Two well-defined classes can be distinguished: one in which the irregularities of the intra-ocular circulation lasted but a short time, and were apparent only at the commencement of menstruation; another, in which they came on a day or two before the flow manifested itself, persisted during its continuance, and lasted a variable time after it ceased. The first class suffered more or less pain at the time these appearances were noticeable, but presented no abnormal nervous symptoms; the other class complained of mental depression, obscure nervous phenomena and pain in the back and thighs.

Cases characterized by anæmia of the intra-ocular structures pursue much the same course and present many of the symptoms observed in cases of congestion persisting throughout the menstrual period. An ophthalmoscopic examination alone can distinguish between them and determine in a given case whether the cerebral symptoms complained of are due to hyperæmia or anæmia of the brain.

There seems to be no causative relation between the amount of blood discharged and the state of the cerebral circulation. An excessive flow may coincide with an extreme degree of intra-cranial hyperæmia, while all the evidences of intra-ocular anæmia may be present in cases where the menstrual discharge is unusually small. The most satisfactory solution is found when we consider the connections and functions of the sympathetic nervous system. It is a well-known fact that an irritation applied at one part of the sympathetic may manifest itself as an alteration of the structure or functional activity of organs but remotely connected with the nervous branches subjected to experiment. The phenomena most apparent are changes in the vascular supply of the part upon which the irritation is reflected, and these changes are of the nature of anæmia and hyperæmia. It is not impossible that some modification of the molecular structure of the tissues is produced primarily to which the hyperæmia or anæmia is secondary. Be that as it may, in a therapeutical point of view it is of the utmost importance to determine which anatomical condition is present, for the treatment appropriate for the one is very detrimental to the other. This is especially so with respect to the brain, for cerebral symptoms almost precisely similar are produced by either hyperæmia or anæmia. The manner in which certain morbid conditions of the uterine organs produce modifications of the cerebral circulation is inexplicable upon any other hypothesis, while the clinical fact that such changes are produced, especially in certain menstrual derangements, is susceptible of demonstration by any one who will take the trouble to investigate the subject with the ophthalmoscope.

The mental symptoms due to the deranged nutrition of the brain so produced are manifold, and vary from the merest excitability of manner to the most furious mania; from slight depression of spirits to extreme melancholia, and in more than one case I have known the whole character of the individuals so changed as to lead to the commission of the most vicious and disgusting acts.

It is in this latter respect that these cases acquire importance in a medico-legal point of view, and no expert can form an opinion entitled to any weight who has not carefully examined the patient with the ophthalmoscope before, during and after her menstrual epoch. In this manner, and in this manner alone can the physical condition of the intra-cranial organs, as regards these secondary vascular changes, be determined, and a basis of fact acquired upon which to found a rational opinion as to the responsibility or irresponsibility of an accused person.—*Boston Medical & Surgical Journal.*

